# P-107 - ASSESSING THE IMPACT OF OPERATIONAL CHANGES ON THE RELIABILITY OF WASTEWATER COVID 19 SURVEILLANCE DATA IN SCOTLAND, 2023-2025

**DOI:** 10.1093/pubmed/fdag046.098

**Published:** 2026-07-09

**Authors:** Miss Carrie Turner, Dr Elizabeth Dickson, Dr Annemarie van Heelsum, Dr Kate Templeton, Dr Kimberly Marsh, Mr Allan McLeod

**Affiliations:** Public Health Scotland; University of Glasgow; Public Health Scotland; Public Health Scotland; Public Health Scotland; NHS Lothian; Public Health Scotland; Public Health Scotland

## Abstract

**Background:**

Surveillance of COVID-19 in wastewater was established in Scotland in June 2020 to support the pandemic response to provide population-level trends of activity through Polymerase Chain Reaction (PCR) testing of water samples from across the national sewage network. More recently, the programme has undergone several operational refinements, including the relocation in July 2024 to a new testing laboratory accredited for molecular methods and the introduction of more streamlined sampling in July 2025, reducing the avg. number of weekly samples from 200 to 114.

**Objectives:**

We examined the impact of the move to a new testing laboratory that holds accreditation for molecular methods and the reduction in the number of samples to determine whether these changes impacted the quality and reliability of the data from the surveillance system

**Methods:**

COVID-19 RNA viral detections in wastewater from 1 August 2023-15 October 2025 were analysed using a longitudinal, interrupted time series method. Given the lack of seasonality and possible autocorrelation, an Auto Regressive Moving Average (ARMA) model was applied, with intervention points (step and pulse functions) determined using Auto-Correlation Function (ACF) and Partial Auto-Correlation Function (PACF) plots to assess model fit. Final model selection was based on stepwise regression using Akaike & Bayesian Information Criteria (AIC & BIC) to determine goodness of fit. Robustness checks and counterfactuals were performed to validate results.

**Results:**

A significant but short-term (2 weeks) increase in RNA viral detection levels was observed following the laboratory change (CI: 30.96, 99.07, p = 0.00018). No impact was observed following the introduction of more streamlined sampling (CI: -28.057, 18.86, p = 0.7008).

**Conclusions:**

Despite a temporary impact following the changes in laboratory this quickly returned to expected levels. Our results show that there have been no long-term impacts from operational changes on the quality of the surveillance system

